# Quality evaluation of chicken soup based on entropy weight method and grey correlation degree method

**DOI:** 10.1038/s41598-024-61667-2

**Published:** 2024-06-06

**Authors:** Zhongwen Cao, Tanglei Zhang, XiKui Tong

**Affiliations:** 1 College of Tourism and Culinary Science, Yangzhou University, Yangzhou, 225000 China; 2 Department of Cooking, Yangzhou Tourism Business Vocational School, Jiangsu Union Technical Institute, Yangzhou, 225000 China

**Keywords:** Chicken soup, Objective quality evaluation, Correlation analysis, Entropy weight method, Grey correlation degree, Health care, Engineering

## Abstract

This study aimed to develop an assessment framework for evaluating the quality of different chicken soup variants. Three types of chicken soup, traditional chicken soup (TCS), concentrated chicken soup (CCS), and blended chicken soup (BCS), were prepared and analyzed for various physicochemical parameters, including gross protein content, crude fat content, pH level, solid content, viscosity, and chromatic aberration value. Sensory evaluation was also conducted to assess overall quality. Correlation analysis helped identify three key evaluation indicators: gross protein content, L* value (lightness), and b* value (chromatic aberration). The weight assigned to gross protein content was the highest using the entropy weight method (EWM). Moreover, the grey correlation degree method was comprehensively applied to evaluate the chicken soup's quality. This analysis identified TCS and CCS as varieties with superior overall quality, showing a positive correlation with sensory evaluation, consistent with the results of nuclear magnetic resonance (NMR) used in this paper. These results provide theoretical support for assessing comprehensive quality and selecting chicken soup varieties.

## Introduction

Traditional chicken soup, renowned for its aromatic and distinctive taste, is a stple ingredient in numerous culinary creations and savory dishes^1^. Given the hectic pace of modern life and the pivotal role of chicken soup, its industrialization has emerged as an inevitable trend^2^. Industrially produced chicken soups currently available in the market primarily comprise concentrated chicken soup, typically derived from boiling deboned chicken frames sourced from commercially raised chickens^2,3^. Another variant includes blended chicken soup, crafted utilizing concentrated chicken sauce.

In their study, Takakura et al. conducted aroma extract dilution analysis, revealing methylpyrazine, 2-ethyl-4-methylthiazole, 3-(methylthio)propanal, and. (E, E)-2,4-decadienal as the predominant aroma compounds in traditional chicken soup^4^. Defatted freeze-dried chicken soup made from native Korean chickens demonstrated qualitative superiority owing to improved nutritional and taste-related attributes compared to commercial broilers^5^. Enzymatic hydrolysis pretreatment enhances the amino acid content, protein solubility, and physicochemical quality of chicken soup^6,7^. Additionally, research conducted by Zhang et al. demonstrated that chicken breast meat contributed significantly higher levels of umami taste to chicken soup when compared to other cuts^2^. Factors such as stewing time, cooking techniques, and methods impact chicken soup's chemical composition and sensory attributes^8–12^. There are significant differences in quality and flavor between traditional and industrially produced chicken soup.

Beyond the transfer of chemical components from the chicken carcass, such as proteins, fats, carbohydrates, and minerals, chicken soup contains over 300 small molecular compounds, with 91 recognized as taste-active compounds^13^. Moreover, the physical properties of chicken soup, encompassing pH, particle size, color, and viscosity, play a crucial role in shaping its sensory attributes^14^.

The intricate interplay among diverse chemical components and taste compounds, coupled with numerous influencing factors throughout breeding, processing (including cooking mode and time), and storage, pose challenges in accurately evaluating and distinguishing the quality of various chicken soups.

The objective examination of chicken soup often involves expensive and sophisticated installations such as electronic tongues, liquid chromatography methods, nuclear magnetic resonance (NMR), gas chromatography-mass spectrometry, and others^13^.

The sensory evaluation uses the human tongue as a detector and offers direct, unique, and intuitive taste information for chicken soup. While being a rapid method, it is inevitably susceptible to the influence of physiological, physical, psychophysical, and other factors inherent to the panelists^13^. A model has been developed to establish an association between flavor-active compounds ((E)-2-nonenal, (Z)-2-decenal, (E, E)-2,4-decadienal, 1-pentanol, 2-undecanone, and one unknown compound) and sensory data, enabling the preliminary screening of flavor-related compounds in chicken soup^14^. The detection of these compounds was achieved using expensive and complex techniques such as GC–MS and GC–MS/O.

Chemometric correlation analysis is widely utilized in food quality assessment research as it offers a robust approach to comprehensive quality evaluation^15^. The entropy weight method (EWM) is frequently employed in objective assignment methods, enabling the direct derivation of weights for multiple indicators. This method calculates these weights based on a minimal dataset, thereby minimizing the influence of human subjective factors^16^. Grey correlation analysis, rooted in grey system theory, facilitates the quantitative comparison of variables exhibiting dynamic changes. This method reveals strong regularities within the sequences by cumulatively generating random sequences, allowing for a reasoned assessment of each variable. Grey correlation analysis has gained prominence in recent years as it can comprehensively reflect varietal differences in small local samples. It has been applied to evaluate the quality of various products^17^.

This study aimed to utilize correlation analysis to identify significant factors among the numerous nutritional and chemical parameters associated with chicken soup. The weights of these factors were determined using the EWM, and a rating system based on the grey correlation degree was established, which would be compared with the result of the method for NMR. The information obtained from this study serves as a theoretical basis for further analysis in this field.

## Material and methods

### Preparation of chicken soup

This study classified chicken soup into three types: traditional chicken soup (TCS), concentrated chicken soup (CCS), and blended chicken soup (BCS). The raw materials for these three types of chicken soup were obtained from native chickens, commercial condensed chicken soup, and chicken sauce purchased from a supermarket in Yangzhou, Jiangsu, China. The experimental samples of chicken soup were prepared after processing the raw materials. Table 1 presents the essential details of the raw materials, including product categories and brands.

**Table 1:** Basic information of raw materials and soups.

Number	Type	raw material	Brand
1	TCS	Two-year-old Sanhuang chicken	
2	CCS	Condensed chicken soup	Wu Taipo
3	CCS	Condensed chicken soup	Si Yunsheng
4	BCS	Soup treasure	Knorr
5	BCS	Chicken sauce concentrate	Totole

Sanhuang chickens, aged two years, were first eviscerated and cleansed to prepare the chicken soup. The carcass was segmented into 3–5 cm pieces using a kitchen knife. These segments were briefly blanched for five minutes, then cooled under running water and drained. The chicken pieces were amalgamated with ginger (0.5% of the chicken's weight) and shallots (0.8%) in a cooking vessel. This concoction was brought to a boil, carefully removed surface impurities, and the mixture was allowed to simmer for 60 min. Salt was incorporated at a concentration of 0.7% towards the end of cooking. Following natural cooling to ambient temperature, the soup was meticulously strained three times through a silk cloth to ensure clarity. The supernatant layer of chicken fat was removed, and the clear soup was reserved for subsequent analysis.

Wu Taipo's and Shi Yunsheng's concentrated chicken soup were individually heated to boiling on an induction stove (CB2007; AUX, China), then cooled naturally to room temperature. The soups were subsequently filtered using a silk cloth, and the top layer of chicken oil was skimmed off. The resulting supernatant portions were carefully collected and stored in separate containers as samples for further use.

Knorr soup treasure and Totole chicken sauce concentrate were prepared by mixing them with distilled hot water at a concentration of 50 mg/mL. The mixture was thoroughly stirred and allowed to cool naturally to room temperature.These prepared samples were then set aside for further use.

Wu Taipo's concentrated chicken soup, Shi Yunsheng's concentrated soup, Knorr soup treasure and Totole chicken sauce soup were named WCCS, SCCS, kBCS, and TBCS, respectively.

### Chemical reagent

Ten analytical reagents, namely copper sulfate, potassium sulfate, sodium hydroxide, p-nitrophenol, sodium acetate, sodium acetate anhydrous, 37% formaldehyde, acetylacetone, petroleum ether, and anhydrous ether, along with two guaranteed reagents, sulfuric acid and acetic acid, were purchased from Shanghai Anpu Biotechnology Co., Ltd. (Shanghai, China). All of these reagents were of analytical grade.

### Proximate composition determination

The gross protein and crude fat content were determined using the Kjeldahl and Soxhlet extraction method^18^, respectively.

To determine the solid content of the chicken soup, as previously reported^11^with slight modifications, a crucible was dried to a constant weight. A precise 10 g sample of the soup was dried in an oven (DHG-9050A; Shanghai Pudong Rongfeng Scientific Instrument Co., Ltd., China) at 100–105℃ until a constant mass was obtained. The ratio of the solid content was calculated using the following formula:

1 $${\text{y}}=({m}_{2}-{m}_{1})/10\times 100\mathrm{\%}$$

where y: the ratio of the solid content, ${m}_{1}$: the mass of the crucible after constant weight (g), ${m}_{2}$: the mass of the crucible and residue after constant weight (g).

### Physical parameters determination

The pH value was measured by immersing a combined glass electrode of a digital pH meter (PHS-3C; Kuosi, China).

The color difference of the chicken soup was measured with a colorimeter(CR − 400; Kono Minolta, China), which recorded L* (brightness), a* (redness), and b* (yellowing) for each of the five samples. The color measurements followed the method described by Zou et al.^10^.

The viscosity of the chicken soup was measured using a viscometer (NDJ-1E; Shanghai et al., China) for each sample. The viscosity values were measured following the procedure outlined by Zou et al.^10^. All measurements were run in triplicate.

### Sensory evaluation

Palatability tests were carried out on four items: color/appearance (30), aroma/flavor (20), taste (30), and oil film (20) for soup samples.The total score was out of 100. A taste panel comprised twenty trained judges, including professional students (five females and five males, who were culinary students well-versed in chicken soup, evaluated the samples) and ten ordinary persons (six females and four males aged between 18 and 30 years). The judges were instructed to carefully read the questionnaires and understand the meaning of each attribute. They were not permitted to discuss their scores during the evaluation sessions. The final result was determined by averaging the scores for each attribute.

### Correlation analysis

By analyzing the correlation between physicochemical parameters and sensory evaluation results, we identified the critical indicators of chicken soup that are both easily measurable and strongly correlated with sensory evaluation. These core indicators were then utilized in the entropy weighting method.

### Entropy weighting analysis

The following steps can calculate entropy weights^19^. The first step is dimensionless data processing.

2 $$X_{i} j^{*} = (x_{i} j - x_{m} in)/(x_{m} ax - x_{m} in)$$

Where ${x}_{ij}$: the physicochemical parameter of each sample, $i$: the number of samples, in this study, $i=\mathrm{1,2},\mathrm{3,4},5, j=\mathrm{1,2},3,\cdots$ *n*, $j$: the number of the core indicators, ${x}_{min}$: the minimum value of the jth indicator, ${x}_{max}$: the maximum value of the jth indicator, and ${{\text{X}}}_{{\text{ij}}}^{*}$: the value of dimensionless processing relative to ${x}_{ij}$.

Step two: Calculate the entropy value of the jth indicator

3 $${Y}_{ij}={X}_{ij}^{*}/\sum_{i=1}^{5}{X}_{ij}^{*}$$

where ${Y}_{ij}$: the proportion of jth indicator of the ith sample,

Then

4 $${e}_{j}=-1/{\text{ln}}5\sum_{i=1}^{5}{Y}_{ij}{\text{ln}}{Y}_{ij}$$

where ${e}_{j}$: the entropy value.

Step three: Calculate the weight coefficient of the jth indicator;

5 $${W}_{j}=(1-{e}_{j})/\sum_{j=1}^{n}(1-{e}_{j})$$

where ${w}_{j}$: the weight coefficient of the jth indicator, *n*: the result of the aforementioned correlation analysis, which means the number of core indicators.

### The gray correlation degrees analysis

The following steps can calculate the gray correlation degrees of the parameters^19^. The first step is dimensionless data processing.

6 $${X}_{ij}={x}_{ij}/{R}_{j}$$

where ${X}_{ij}$: the value of dimensionless processing relative to ${x}_{ij}$, ${R}_{j}$: the optimal value of the jth indicator, which is from the Traditional chicken soup according to the result of sensory evaluation in this work.

Step two: Calculate the grey correlation coefficients:

7 $${\varepsilon }_{i}\left(j\right)=({min}_{i}{min}_{j}{\Delta }_{i}\left(j\right)+\rho {max}_{i}{max}_{j}{\Delta }_{i}\left(j\right))/({\Delta }_{i}\left(j\right)+\rho {max}_{i}{max}_{j}{\Delta }_{i}\left(j\right))$$

where ${\varepsilon }_{i}\left(j\right)$: the grey correlation coefficient,

8 $${\Delta }_{i}(j)=\left|1-{X}_{ij}\right|$$

$\uprho$: resolution coefficient ($\uprho =0.5$ in this study).

Step three: Calculate the grey correlation degree:

9 $${r}_{i}^{*}={\sum }_{j=0}^{n}{w}_{j}{\varepsilon }_{i}\left(j\right)$$

where ${r}_{i}^{*}$: the grey correlation degree of the ith sample.

### NMR transverse relaxation measurements

The samples were transferred to a unique NMR tube. The G50-Probe probe of the NMR analyzer (AccuFat-1050, McCormick Corporation, Nanjing, China) was selected and calibrated. The CPMG one-dimensional pulse sequence was used to measure the samples, and each sample was repeated three times. The sample acquisition parameters were set as follows: Receiver Gain = 20 dB, Sampling interval = 4 ms, Points = 5000, Scans = 16, Re cycle delay = 15 s. After the measurement, the results were inverted using the analysis software (Version 4.02, McCormick Corporation, Nanjing, China).

### Statistical analysis

Correlation analysis was conducted using the SPSS (version 20.0 SPSS Inc., Chicago, USA). Entropy weights were calculated using Microsoft Excel 2010. Subsequently, gray correlation analysis was performed based on the obtained weights. All analyses were performed in triplicate to ensure accuracy and reliability.

## Results and discussion

### Proximate composition analysis

The gross protein content of chicken soups exhibited significant differences, as shown in Table 1. The gross protein content in chicken soup primarily consists of.

chondroitin, collagen, and free amino acids^20^. Among the samples, TCS had the highest gross protein content. This finding is consistent with Qi et al.^8^, who reported that prolonged stewing time significantly increased soups' protein, fat, and solid contents. Extended stewing duration can hydrolyze proteins and increase carbohydrate content in the soup, leading to Maillard reactions^21^.

In this paper, the measurement error of protein is significant, which may be due to the following reasons: the samples may not be converted to the same degree during the digestion process, resulting in an inconsistent estimation of the nitrogen content; during the distillation process, there may be volatile loss of ammonia, resulting in an inconsistent estimation of the nitrogen content; during the titration process, the accuracy of the titration endpoint may be affected by the subjective judgment of the change in the color of the indicator; and all these reasons may result in a significant error in the calculation of the final protein.

Furthermore, Wattanachant et al. observed that native chicken muscles had higher protein content compared to broiler muscles^22^, which could contribute to the higher protein content in traditional chicken soup made from native chicken compared to that made from commercial broilers.

Fat in soup serves as an essential reservoir for aroma compounds^23^. The crude fat content of TCS was significantly higher (P < 0.05) than that of CCS and BCS, with SCCS having a zero fat content. This finding contradicts the conclusion that native chicken muscles contain lower fat than broiler muscles^22,24^. This may be because the high-fat content of the soup promotes its emulsifying ability because of high-protein content^25^.

While fat contributes to the pleasant flavor of chicken soup, excessive fat content can lead to a greasy taste and overly oily flavor, affecting the sensory quality of the soup^26^. Moreover, excess fat has implications for health considerations. CCS generally has low-fat content due to fat removal during the production process^27^, reducing solids content. In the case of SCCS, fat has been completely removed during the production process, resulting in a clear chicken soup without fat, as indicated by its packaging.

On the one hand, BCS had not been stewed for an extended period. On the other hand, the chicken sauce primarily comprises various additives listed in their ingredients, with a smaller amount of chicken meat, which resulted in BCS having the lowest gross protein, solid content, and lower fat content.

The solid content is an essential indicator for assessing soup quality and reflects the overall dissolution of nutrients and flavor substances^28^. These substances primarily originate from amino acids, minerals, glycogen, vitamins, and other soluble substances released from chicken meat tissues^29^. Table 2 shows that TCS had the highest solid content at 1.83%, while BCS had the lowest. This result aligns with the crude protein and gross fat content levels, mainly influenced by the stewing time^11^.

**Table 2:** The physicochemical properties of different chicken soups.

Samples	Gross Protein (mg/100 mL)	Crude fat (mg/100 mL)	Solid (%)	Viscosity (cps)	pH	L*	a*	b*
TCS	0.85 ± 0.15^a^	1.69 ± 0.01^a^	1.83 ± 0.02^a^	0.97 ± 0.02^e^	6.4 ± 0.1^b^	42.82 ± 0.33^a^	− 0.21 ± 0.02^a^	7.84 ± 0.02^a^
WCCS	0.72 ± 0.18^b^	1.42 ± 0.02^b^	1.78 ± 0.04^a^	4.07 ± 0.03^b^	6.8 ± 0.2^a^	42.8 ± 0.46^ab^	− 0.31 ± 0.03^b^	7.81 ± 0.08^a^
SCCS	0.68 ± 0.24^b^	0 ± 0^e^	1.17 ± 0.05^b^	3.31 ± 0.04^c^	6.2 ± 0.1^b^	40.52 ± 0.10^c^	− 0.15 ± 0.03^a^	4.38 ± 0.03^c^
KBCS	0.24 ± 0.09^d^	0.68 ± 0.01^c^	1.08 ± 0.01^b^	4.2 ± 0.14^a^	6.1 ± 0.1^b^	41.98 ± 0.44^b^	− 0.45 ± 0.01^c^	4.34 ± 0.03^c^
TBCS	0.47 ± 0.20^c^	0.42 ± 0.01^d^	0.92 ± 0.02^c^	1.18 ± 0.24^d^	6.2 ± 0.1^b^	40.94 ± 0.32^c^	− 0.38 ± 0.01^bc^	6.87 ± 0.02^b^

Data are expressed as mean ± SEM. Mean values with different superscript letters [a (the highest value)—e (the lowest value)] in the same row are significantly different at (p < 0.05).

### Physical parameters analysis

The viscosity of the five samples is shown in Table 2. The results indicated significant differences (p < 0.05) not only between different types but also among chicken soups of the same type. Factors such as temperature, concentration, pH, the interaction between protein molecules, fat particles, and particle size affect viscosity^12^. KBCS might contain smaller particles, resulting in higher viscosity values^30^, making its viscosity the highest. The viscosity of WCCS is greater than that of SCCS, possibly due to the order of taste compound content^12^, as SCCS removed fat, which may have removed other substances. However, TCS had the lowest viscosity due to its highest fat content, and the presence of fat can cause emulsification effects, thus reducing viscosity^10^.

pH plays a crucial role in determining chicken soup flavor^31^. pH range of 6.16–6.38 can create a stable emulsion in chicken soup^27^. According to Table 2, the pH of TCS is lower than that of WCCS, which results from the higher muscle pH in commercial broilers compared to native chickens, as determined by Wattanachant et al.^22^. Furthermore, as stewing time increases, lactic acid accumulates, leading to increased acidic substances and a significant decrease in pH^32^. The difference in pH between WCCS and SCCS can be attributed to an enzymatic hydrolysis process involved in the preparation of SCCS. This process causes H + ions to dissociate from free amino groups into the hydrolysis medium during enzymatic cleavage of peptide bonds, thereby affecting the pH difference^33^. As for BCS, the mixing process likely influences the pH value, as it falls within the range that enhances taste^31^. Apart from.

WCCS, there are minimal variations in pH values among the different samples, indicating that pH cannot serve as an indicator for distinguishing between types of chicken soup.

Color is an essential factor in evaluating soup quality^11^. Both TCS and WCCS exhibited the highest L* value, according to Table 2, attributed to their higher content of crude fat, gross protein, and solids^10^. The a* value for all soups was smaller than the b* value, indicating that the yellow color dominates, which aligns with the findings of Zou et al.^10^. In Table 2, the b* value for TCS was higher compared to WCCS and SCCS, and the a* value for TCS was also higher than that of WCCS. In contrast, the b* value showed no significant difference between TCS and WCCS. These results are generally consistent with previous studies showing that native chicken muscles exhibit lighter, redder, and yellower characteristics compared to commercial broiler chicken muscles^22^.

### Sensory evaluation

Table 3 shows that TCS obtained the highest score while BCS received the lowest. Relevant studies have shown that the sensory quality of chicken soup is significantly influenced by factors such as gross protein content, crude fat content, and solid content^8,11^. TCS had the highest gross protein, crude fat, and solids levels, resulting in an intense aroma and sweet taste^9^.

**Table 3:** Statistical result of sensory evaluation.

Sample number	TCS	WCCS	SCCS	KBCS	TBCS
Score	96.9	92.4	90.5	82.2	80.3

The storage duration of CCS affects its sensory properties due to the chemical and biochemical changes caused by microorganisms during the storage process, leading to a deterioration in the sensory characteristics of the soup^13^. Ashfaq revealed that storage leads to a significant decline in overall acceptability^34^. BCS, which is made from additives, spices, chicken oil, and small pieces of chicken meat, exhibited the lowest sensory ratings.

### Correlation analysis

As presented in Table 4, there was a strong correlation between the sensory scores and the gross protein content, L* value, and b* value of the chicken soup. As mentioned earlier, the crude protein content plays a crucial role in the taste, aroma, and nutritional value of chicken soup, while the L* and b* values significantly impact the color, which directly influences consumers' sensory perception and is an essential factor in determining chicken soup quality. Although the correlation between sensory scores and fat content, as well as sensory scores and solid content, was not statistically significant, the correlation coefficients were large, indicating that the influence of fat and solid content on sensory characteristics was perceivable but weaker compared to the influence of protein content, L* value, and b* value. Additionally, there was a strong correlation between L* values and solid content and between crude fat and solid content. Therefore, crude protein, L* value, and b* value were identified as representative indicators of chicken soup.

**Table 4:** Correlation analysis.

Division	Crude fat	Gross protein	Solid	Viscosity	pH	L*	a*	b*	Sensory scores
Crude fat	1								
Gross protein	0.862	1							
Solid	0.989*	0.772	1						
Viscosity	− 0.062	− 0.373	− 0.04	1					
pH	0.691	0.611	0.803	0.168	1				
L*	0.882	0.256	0.950*	0.15	0.687	1			
a*	0.869	0.835	0.423	− 0.237	0.198	− 0.197	1		
b*	0.57	0.907*	0.637	− 0.448	0.761	0.971*	0.048	1	
Sensory scores	0.869	0.965*	0.901	− 0.368	0.356	0.968*	0.132	0.975*	1

**p < 0.01, *P < 0.05.

### Indicator weights assigned by the entropy weighting method

Hence, Eq. 2–5 were utilized to compute the information entropy value and indicator weights, as displayed in Table 5. The weight assigned to gross protein is the highest, indicating that gross protein holds the utmost significance for chicken soup.

**Table 5:** Information of the entropy value and the weight coefficient of indicators.

Indicator	Entropy value	Weight coefficient
The gross protein	0.989344	0.364661
L*	0.991108	0.304287
b*	0.990326	0.331052

### Results of grey correlation analysis

The gray correlation coefficients for the three indicators of various chicken soups were computed using Eqs. 6,7. By referring to Table 5, Table 6, and Eq. 8, the weighted correlations of the different chicken soups were determined, as presented in Table 7.

**Table 6:** The grey correlation coefficient of different chicken soups.

Type of chicken soup	The gross protein	L*	b*
TCS	1.0000	0.3905	0.5842
WCCS	1.0000	0.3878	0.6259
SCCS	1.0000	0.4888	0.7183
KBCS	1.0000	0.5174	0.7887
TBCS	1.0000	0.4906	0.7533

**Table 7:** Weighted correlation and comprehensive ranking of different chicken soups.

Type	Weighted correlation	Comprehensive ranking
TCS	0.7832	1
WCCS	0.7512	3
SCCS	0.7633	2
KBCS	0.6899	4
TBCS	0.6769	5

The higher the weighted correlation, the more closely it aligns with the quality of the ideal variety. The reason for the similar sequences of CCS and TCS compared to BCS is due to the proximity of their raw materials.

### Results of NMR transverse relaxation measurements

Supplementary table 1 shows the NMR data of five different chicken soup products. The relaxation time is related to the mobility of the hydrogen proton, with longer relaxation times corresponding to more mobile hydrogen protons and shorter relaxation times corresponding to less mobile hydrogen protons. Due to the different degrees of freedom of water in different chicken soup samples and their different chemical environments of hydrogen protons, the NMR data have different *T*_2_ value distributions and amplitude values. In supplementary table 1, TCS and WCCS had a signal response from 12.16 to 24.17 ms, which may be due to the high content of hydrated proteins and oil components in TCS and WCCS. Comparison of the NMR relaxation properties allowed the five chicken soup products to be divided into two groups, i.e., TCS and WCCS as one group, and SCCS and BCS as one group. The NMR test data does not directly distinguish between the five types of chicken soup.

### NMR data combined with PCA for the differentiation of different chicken soup

Since the NMR data could not accurately distinguish the different chicken soups, it is necessary to analyze the characteristic components of the different chicken soups further using principal component analysis (PCA). Principal Component Analysis (PCA) extracts pertinent insights regarding the variables impacting sample similarity and dissimilarity. It facilitates dimensionality reduction, enhancing analysis efficiency by consolidating numerous indicators into a handful of composite ones while minimizing information loss^19^. The relaxation time *T*_2_ and peak area *S* of each chicken soup sample were selected for PCA: relaxation time *T*_21_ (*u*_1_), peak area *S*_1_ (*u*_2_), relaxation time *T*_22_ (*u*_3_), peak area *S*_2_ (*u*_4_), relaxation time *T*_23_ (*u*_5_), peak area *S*_3_ (*u*_6_), and the variance and cumulative contribution of the eigenvalues were calculated and the number of principal components was extracted according to the cumulative contribution of ≥ 80%. The number of components was extracted according to the principle that the cumulative contribution rate was ≥ 80%. A higher cumulative contribution indicates that the chosen principal components preserve most information or variance in the original data set, thus minimizing information loss throughout the dimensionality reduction process^35^.

As shown in supplementary table 2, two principal components were obtained in this study, and their corresponding variance contributions were 52.688% for principal component 1 (*PC*_1_) and 30.949% for principal component 2 (*PC*_2_). The cumulative contribution rate of the above two principal components was 83.617%, fully reflecting all the original information of the six variables of the measured chicken soup products by NMR.

As shown in supplementary table 3, *PC*_1_ was significantly correlated with relaxation time *T*_21_ , peak area *S*_1_, peak area *S*_2_, relaxation time *T*_23,_ and peak area *S*_3_. *PC*_2_ was significantly correlated with relaxation time *T*_22_ , peak area *S*_2,_ and relaxation time *T*_23_.

Based on the eigenvalues in supplementary table 2 and the component matrix in supplementary table 3, the model for the two principal components ( ${F}_{1}$ and ${F}_{2}$) can be calculated as follows:

10 $${F}_{1}=0.531{u}_{1}+0.534{u}_{2}-0.131{u}_{3}-0.321{u}_{4}-0.435{u}_{5}+0.329{u}_{6}$$

11 $${F}_{2}=0.101{u}_{1}+0.062{u}_{2}+0.705{u}_{3}+0.6001{u}_{4}-0.318{u}_{5}+0.165{u}_{6}$$

From the eigenvalues in supplementary table 2, the principal component expression of the total ($F$) is calculated as:

12 $$F=0.630{F}_{1}+0.370{F}_{2}$$

As a result, the main ingredients of 5 kinds of chicken soup were ranked, as shown in supplementary table 4. It can be seen from supplementary table 4 that the TCS principal component score is the highest, followed by CCS, and the BCS comprehensive score is the lowest, which is consistent with the ranking result of Table 7.

### Model validation

To validate the accuracy of the assessment model developed using EWM and the gray correlation method; a regression analysis was performed on the weighted correlation results (denoted as x in Table 7) and the sensory scores (denoted as y in Table 3). The analysis yielded a linear equation y = 146.43x + 18.858 with an R-squared value of 0.9565. The elevated correlation coefficient suggests that the model is well-suited for evaluating the quality of chicken soup. Moreover, the parameters involved in this model are more straightforward to detect and more convenient to use than the model proposed by Zhang et al.^14^, which measured many indicators, applied complex and expensive GC–MS and GC–MS/O systems, and ultimately modeled the objective data by correlating it with the subjective data, this paper has less objective data and is easy to detect, less costly, and more efficient.

## Conclusion

In conclusion, this study examined the variations in physicochemical parameters of chicken soups associated with sensory quality. By utilizing crude protein content, L* value, and b* value as representative parameters derived from correlation analysis, the model constructed using EWM and the gray correlation method effectively captures the quality evaluation of TCS, CCS, and BCS. The method provided herein allows for a more straightforward differentiation of different chicken soups, involves fewer assays, and is easy to detect.

Compared with the traditional instrumental determination and sensory evaluation,this paper reduces the physicochemical testing items and inaccuracy of human subjective judgment. It provides a new reference for distinguishing the quality of many food products, including chicken soup. Indeed, the quality of chicken soup also varies due to other physicochemical indicators, such as the content of flavor substances. In subsequent research, it is necessary to include more evaluation indicators to provide better theoretical guidance for distinguishing the quality of chicken soup.

### Supplementary Information

Supplementary Tables.

## Data Availability

The data presented in this study are available on request from the corresponding author.
