# Supplementary material for: Quality evaluation of chicken soup based on entropy weight method and grey correlation degree method
Source: Sci Rep. 2024 Jun 6;14:13038. doi: 10.1038/s41598-024-61667-2 (PMC11156652; doi:10.1038/s41598-024-61667-2)
Supplement: Supplementary file 1 — Supplementary Tables. [file 41598_2024_61667_MOESM1_ESM.docx]

| Relaxation parameters | TCS | WCCS | KBCS | TBCS | SCCS |
| --- | --- | --- | --- | --- | --- |
| Relaxation  time *T*_21_(ms) | 24.17±2.11^a^ | 12.16±1.03^b^ | - | - | - |
| Peak area *S*_1_ | 2.44±0.12^a^ | 0.63±0.02^b^ | - | - | - |
| Relaxation time*T*_22_(ms) | 225.64±3.55^b^ | 162.95±4.28^c^ | 391.64  ±2.78^a^ | 116.15  ±1.52^d^ | 165.67  ±2.16^c^ |
| Peak area*S*_2_ | 40.75±1.85^bc^ | 20.21±0.93^d^ | 121.33  ±4.23^a^ | 37.42  ±1.56^c^ | 45.48  ±2.13^b^ |
| Relaxation time*T*_23_(ms) | 1700.01±15.23^d^ | 2186.42  ±23.45^b^ | 2180.34  ±14.88^b^ | 2365.18  ±18.45^a^ | 2053.34  ±19.52^c^ |
| Peak area*S*_3_ | 2952.01±23.12^ab^ | 3209.31  ±32.45^a^ | 2744.76  ±30.98^b^ | 2223.51  ±29.82^c^ | 3172.23  ±42.24^a^ |

Supplementary table 1. NMR nuclear magnetic data of 5 kinds of chicken soup products. Different lowercase letters in the upper right corner of the same column indicate a significant difference between values (*P*<0.05).

| Principal component | Principal component characteristic root | contribution  rate(%) | Cumulative contribution  rate(%) |
| --- | --- | --- | --- |
| 1 | 3.160 | 52.688 | 52.688 |
| 2 | 1.857 | 30.949 | 83.617 |

Supplementary table 2. Principal component characteristic root and contribution rate

| Element | *PC*_1_ | *PC*_2_ |
| --- | --- | --- |
| *T*_21_ | 0.944 | 0.138 |
| *S*_1_ | 0.949 | 0.085 |
| *T*_22_ | -0.232 | 0.961 |
| *S*_2_ | -0.571 | 0.818 |
| *T*_23_ | -0.805 | -0.433 |
| *S*_3_ | 0.584 | 0.225 |

Supplementary table 3. Compositional matrix of NMR data for five chicken soup products

| Samples | *F*_1_ | *F*_2_ | *F* | Ranking |
| --- | --- | --- | --- | --- |
| TCS | 172.26 | 132.36 | 157.50 | 1 |
| WCCS | 44.91 | -37.41 | 14.45 | 3 |
| SCCS | 77.20 | 14.54 | 54.02 | 2 |
| KBCS | -174.92 | 108.44 | -70.08 | 4 |
| TBCS | -367.12 | -280.91 | -335.22 | 5 |

Supplementary table 4. Principal component scores, comprehensive scores, and rankings of five chicken soup
